# Do Religiosity and Ethnocentrism Influence Indian Consumers’ Unwillingness to Buy Halal-Made Products? The Role of Animosity Toward Halal Products

**DOI:** 10.3389/fpsyg.2022.840515

**Published:** 2022-05-06

**Authors:** Meng Tao, Eva Lahuerta-Otero, Faizan Alam, Jehad Saleh Aldehayyat, Md. Rashid Farooqi, Peng Zhuoqun

**Affiliations:** ^1^School of Business Administration, Dongbei University of Finance and Economics, Dalian, China; ^2^Instituto Multidisciplinar de Empresa, Universidad de Salamanca, Salamanca, Spain; ^3^College of Business Administration and Economics, Al-Hussein Bin Talal University, Ma’an, Jordan; ^4^Department of Management and Commerce, Maulana Azad National Urdu University, Hyderabad, India; ^5^School of Tourism Management, Dongbei University of Finance and Economics, Dalian, China

**Keywords:** boycott attitude, religiosity, halal product animosity, Indian Hindus–Muslims, unwillingness to buy

## Abstract

The purpose of this research is to assess the relationship between Indian (non-muslims) consumers’ animosity toward halal products and their unwillingness to buy halal products. Moreover, we seek to investigate boycott attitudes, religiosity, ethnocentrism, and patriotism as drivers of consumer animosity and reluctance to purchase halal products. Consumer animosity toward halal products has received some attention in marketing research, and we aim to further explore animosity regarding the halal label. We collect online survey responses from 512 Indian consumers and analyze the data using SmartPLS3 software. Findings show a positive impact of boycott attitude, religiosity, and ethnocentrism on Indian consumers’ level of animosity and thus their unwillingness to purchase halal products. Furthermore, consumer animosity acts as a mediator of the relationship between boycott attitude, religiosity, ethnocentrism, patriotism, and reluctance to buy halal products. Notably, the findings provide a menu of potential managerial actions to reduce or enhance consumer animosity.

## Introduction

India is a secular nation (Smith, 2015, p. 24) consisting of a large Hindu community and a minority community of different religions. Consequently, Hindu people represent a large share of potential consumers in the Indian market (Deb and Sinha, 2015). Various non-Muslim nations (Berry, 2008; Ayyub, 2015) have a rising requirement for halal food items, which have developed into a growing market (Euromonitor International, 2010). However, in India, halal products are a controversial topic among the Hindu majority, many of whom see the growth of this market as Islamic economic jihad. According to Ayyub (2015), the fast growth of the halal food market is characterized by two factors: first, halal food is known to be safer, cleaner, and tastier (Alam and Sayuti, 2011), and second, halal food has been extensively adopted among the global population through the process of acculturation and assimilation (Ayyub, 2015). Food is an essential element in people’s lives. In many countries, an infinite variety of food is available, and food selection decisions represent today’s consumers’ initial purchase behavior (Jamal and Sharifuddin, 2015; Naeem et al., 2019). Food is a form of calorie intake, but also serves a social and cultural role (Steenkamp, 1993). In today’s global economy, a growing number of customers are resorting to boycotting to demonstrate their disappointment with an organization and its corporate practices (Sen et al., 2001; Klein et al., 2004). When a firm is boycotted, it runs the risk of missing revenue, cash flow, and profits, as well as facing a potential drop in stock price (Farah and Newman, 2010). Consumer boycotts have traditionally been thought of as mass acts of anti-consumerism to implement an operational shift in a corporation’s target market or bring about a societal alteration throughout the whole structure of trade and marketing (Friedman, 1985; Garrett, 1987; Muhamad et al., 2019). Many boycotts of products or corporations have emerged recently as acts of agitation for social, national, or ethical reasons. Some boycotts were seen as a social good, while others developed a bad reputation.

Information can be transmitted almost instantly through the Internet, television, and social media with technologically dynamic telecommunication infrastructure. Many Indians and political figures, including news anchors, criticize halal vendors, Muslims, and tweet #BoycottHalalProducts, #IslamisationofIndia, #Economic-Jihad, and other similar hashtags in an aggressive confrontation between Hindus and Muslims (Hindu Janajagruti Samiti, 2020). Consumers will spontaneously boycott goods, commodities, or practices that they believe are unfair. Furthermore, whenever a crisis occurs between Israel and Palestine (Mostafa, 2018), India and Pakistan (Baba, 2019), or Afghanistan and the United States (Malkasian, 2021), Hindus have engaged in boycotts of corporations that have been proven to endorse or finance the Muslim-majority nations involved in the conflicts (Pew Research Centre, 2013). During the tension between these countries, India’s reaction to such boycotts was especially noticeable. Several controversies arose in India concerning Hindu–Muslim and Israeli–Palestinian conflict-related boycotts, including questions about the campaigns’ efficacy and appropriateness and the boycotts’ actual target (Hamzah and Mustafa, 2019). Despite the prominent media coverage of the boycotts of halal products, mainly during the Hindu–Muslim controversy in India and the Hindu community’s ongoing shutdown campaign targeting halal products.

India is a nation noted for its cultural minorities. Indians are recognized to be very nationalist and have a socialist ethic (Shimp and Sharma, 1987). Indian people, like those in any other developed nation, are patriotic and prefer domestic goods, but still accept products from other countries (Agbonifoh and Elimimian, 1999; Lee et al., 2011). However, some studies indicate that Indians are ethnocentric (Bawa, 2004), and hence, the “made in India” label is standard among them. This context provides an intriguing opportunity to investigate Indian consumers’ attitudes toward halal products. Although boycotts occur across regional lines, races, sects, and societies, most research in this field has focused on the Western context, with a few exceptions. Thus, it is difficult to find research studies on this subject, particularly in Asia and India. As a result of differences in customers’ identities, values, religions, and environments, major consumer culture elements such as consumer ethnocentrism, religiosity, patriotism, animosity toward halal products, and unwillingness to purchase halal goods must be considered.

Shoppers have begun to investigate food products based on a variety of food-related considerations and circumstances. Since many people observe religious principles, religious background is one of the most important elements in food selection and eating. Moreover, the degree to which practitioners of various religious sects adhere to dietary rules differs. For example, in the United States, it has been recorded that 90% of Buddhists and Hindus, 75% of Muslims, and 16% of Jews observe religious dietary rules (Lada et al., 2009). According to Islam, Muslims must eat halal food (Bonne et al., 2007). Halal is an Arabic term meaning “legitimate and permitted by Allah,” while haram means “prohibited or forbidden” (Bonne and Verbeke, 2008; Naeem et al., 2019). Food must be halal, salaries must come from halal markets, and one can only deal with halal trading options. As a result, Muslim customers are required by their faith to only purchase halal products, and thus, they look for the halal logo and halal-labeled goods. These statistics not only support the claim that halal food products are in high demand in both Muslim and non-Muslim countries but also encourage companies around the world to prioritize halal products to gain total market penetration (Ali et al., 2017). Pharmaceutical companies, medicines, protective devices, personal hygiene products, and healthcare facilities are also included in the halal market. Hindu customers are seen halal products as an economic threat to Indian firms, which becomes a severe matter for majority Indian-Hindu against halal products.

This research aims to better understand consumer animosity toward halal products, focusing on particular factors. We focus on boycotts (Klein et al., 1998; Klein, 2002), patriotism (Marinkovic, 2017), consumer ethnocentrism (Shimp and Sharma, 1987; Klein, 2002), and religiosity (Ahmed et al., 2013) as the key factors influencing Indian consumers’ unwillingness to purchase halal goods. In addition, this study identifies explanations for why customers may avoid religiously themed goods (halal-labeled goods). Finally, we look at how Indian customers respond to Muslim-directed halal products in particular.

## Literature Review

When making judgments on goods, consumers will consider the product’s nation of origin (Zdravkovic, 2013). The influence of a product’s country of origin (COO) has been investigated in several product categories and various countries (Khan et al., 2017). One of the key results of these investigations is that the COO provides a framework for distinguishing between similar products. The latest evidence demonstrates that the COO significantly impacts consumers’ unwillingness to buy a specific product (Aichner et al., 2017). In particular, several factors, including patriotism, ethnocentrism, and animosity, have been established as impacting a customer’s use of the COO as an identifier. For example, consumers may have positive feelings associated with purchasing locally produced products and feel irritation with goods from other communities (Sharma et al., 1994; Asseraf and Shoham, 2016), meaning that certain people show an unwillingness to buy depending on a product’s COO.

Shoham et al. (2006) state that animosity’s emotional and societal effect can be discussed within the context of cognitive dissonance theory (Festinger, 1957), which implies that an individual knows their self, their previous behavior, and their beliefs and attitudes. These elements of oneself can be seen as consonant if one component is compatible with another and dissonant if not. For example, the perceived qualities of a halal product or service may be discordant with the beliefs and practices of Indian-Hindu consumers. Due to cognitive dissonance being psychologically unpleasant, consumers tend to avoid it, which negatively affects purchase intentions. Ultimately, research into boycotting can significantly examine the impact of affective and normative animosity. A boycott appears whenever several individuals simultaneously abstain from buying a commodity due to almost the same unethical act or behavior, but not generally for similar reasons (John and Klein, 2003). Consumers engage in boycotts to demonstrate significant disagreement with a corporation or nation’s actions and practices (Shaw et al., 2006). Furthermore, animosity plays an essential role in perceptions toward intervening through boycotting (Smith and Li, 2010). A boycott can, therefore, be an expression of animosity.

### Boycott Attitude

The “Boycott Halal Products” movement appears to be part of a more significant anti-Islam initiative (Menon, 2020). Boycott attitudes by potential consumers are a severe cost for businesses; boycotts can result in a reduction in income and a decrease in overall revenue. Boycotts further have the potential to sabotage the company’s partnerships with its wholesalers. Customers are at the heart of every enterprise, seen as capital to ensure the company’s survival. As a result, the boycott of a certain product affects the product’s business’s market perception. A positive or negative inclination toward anything is referred to as attitude. Boycotting creates a negative perception of goods that ultimately leads to people refusing to purchase them (Bhattacharjee, 2020). It is also important to emphasize that boycotted items may have a detrimental influence on the sale of “non-boycotted” items. When deciding whether to boycott a product, the boycotting customer forms an anti-product stance (Dekhil et al., 2017). In general, boycotting can affect a company’s sales of both boycotted and non-boycotted items and threaten the company’s overall viability.

In the context of India, boycotts against halal products have been encouraged by Hindu organizations. Since India’s population is 15% Muslim and the remaining 85% is non-Muslim, the Hindu Janajagruti Samiti (HJS) has called on the government to end the practice of halal certification and for Indian people to boycott halal-certified goods (Hindu Janajagruti Samiti, 2020). As such, we theorize that:

*H1*: Boycott attitude positively affects Indian consumers’ animosity toward halal products.

### Consumer Ethnocentrism

The term “ethnocentrism” has its roots in sociology. As a concept, it emphasizes individuals who regard their community as the most significant part of their identity and knowingly embrace others who belong to the same culture, while concurrently rejecting those who are ethnically different from them (Booth, 2014). In the Indian context, consumer ethnocentrism is defined as the belief among buyers that buying halal goods is improper or even unethical and harmful to the national economy, for Hindu religion, detrimentally affects local employment, and is unpatriotic (Shimp and Sharma, 1987). According to Sharma et al. (1994), consumer ethnocentrism signifies an individual’s preference for local products over foreign goods. Prior research has shown that countries with strong ethnocentric inclinations are likely to harbor unfavorable perspectives toward foreign products (Park and Yoon, 2017). Ethnocentric consumers show a high preference for domestic products. Indeed, so much so that they often overestimate the product characteristics and demonstrate strong support for local goods (Siamagka and Balabanis, 2015).

It is overwhelmingly assumed that ethnocentrism has a desirable impression on both non-halal domestic products (Shimp and Sharma, 1987; Fernández-Ferrín et al., 2015). However, the role played by ethnocentrism often varies from individual to individual as well as from country to country. Even after recognizing halal goods as higher quality, some exceedingly ethnocentric shoppers only buy non-halal products (Vida and Reardon, 2008). Ethnocentric consumers hold a critical view of halal foreign products and are unwilling to buy them (Ahmed et al., 2013). We therefore establish ethnocentrism as a key factor within the current study, considering India’s history of merging various cultures.

Ethnocentrism thus plays a significant role in influencing the behavior of Indian consumers regarding the purchase of halal products. Generally, ethnocentrism provokes a similar feeling to animosity (Akdogan et al., 2012). As such, we theorize that:

*H2*: Consumer ethnocentrism positively affects Indian consumers’ animosity toward halal products.

### Patriotism

Patriotism can be described as an individual’s affection and concern for a nation, as well as the extensiveness of one’s associations with the nation’s land and flag (Berns, 1997; Marinkovic, 2017). Patriotism is widely acknowledged as including two aspects of an individual’s national sense of belonging: instrumental and sentimental attachment (Meier-Pesti and Kirchler, 2003). Instrumental attachment refers to the privileges that an individual may obtain as a citizen of a particular country—the citizenship that identifies an individual as a part of a specific nation is the most exact illustration of this association (Marinkovic, 2017). In this framework, it can be inferred that this form of attachment is based on an individual’s logical thinking. When personal beliefs align with one’s domestic counterparts, an emotional connection form. This association derives from heritage, culture, devotion to an artistic logo, and an interpersonal reaction to patriotism. Both patriotism and nationalism are usually connected to a positive association with a specific country. Nationalism, however, means one country’s dominance and supremacy over another. While patriotism entails a love of the country, it does not mean national superiority (Van der Toorn et al., 2014). Patriotism embodies a person’s sense of national pride and affinity toward locally manufactured products, i.e., non-halal products. These positive feelings may emerge from an emotional bond connected to the essential nature of the country or product. As such, we hypothesize that:

*H3*: Patriotism positively affects Indian consumers’ animosity toward halal products.

### Religiosity

According to Gbadamosi (2021), religion originates from “re” and “ligare,” or “binding together”; it ties together the material world with the non-material, infinite concept of God. Indian society displays a strong affinity to religion, and their political, cultural, and economic considerations are primarily affected by religious belief. Religious devotion impacts the everyday life of Indian consumers. Hinduism, the dominant religion in Indian society, plays a significant part in family, work, and social relationships (Scroope, 2016). The link between religious beliefs and buying intentions and behaviors is grounded in extant literature (e.g., Delener, 1994; Sood and Nasu, 1995; Choi, 2009; Jianfeng et al., 2009). Sood and Nasu (1995) published one of the earliest studies connecting religious faith to buying behavior. A study conducted by Srivastava (2010) indicates that individual religious belief influences consumers’ purchasing intentions toward foreign and domestic goods in emerging nations, such as India. As such, we hypothesize that:

*H4*: Religiosity positively affects Indian consumers’ animosity toward halal products.

### Consumer Animosity Toward Halal Products and Unwillingness to Buy Halal Products

Several earlier studies show that animosity decreases customers’ willingness to purchase goods from foreign countries (Klein et al., 1998; Nijssen and Douglas, 2004; Ettenson and Klein, 2005; Rose et al., 2009). Calls for the boycott of halal products, especially boycotts of products with the halal logo (thus encouraging animosity toward halal products), have been relatively successful campaigns. Millions of Indian (Hindu) consumers have followed political figures’ and news anchors’ requests and supported non-halal products and services. For example, consumers urged the Indian government to not put the halal stamp in Arabic and to put a non-halal (*Jhatka*) stamp on products for their convenience. Consumers’ negative emotional attitudes toward products or services are known as consumer animosity (Klein and Ettensoe, 1999). Past or current disputes between Hindus and Muslims can result in this deep-rooted anger (Riefler and Diamantopoulos, 2007). Thus, the concept of animosity helps to understand customers’ adverse behavior toward halal products and their unwillingness to purchase halal products.

*H5*: Consumer animosity positively affects Indian consumers’ unwillingness to purchase halal products.

### Mediation Role of Consumer Animosity Toward Halal Products

Religious affirmations may elicit negative sentiments and contribute to a decline in purchases when customers have unfavorable opinions or prejudices against religion (Simonin and Ruth, 1998). If consumers do not belong to the religion, they may see goods with religious affirmations as distinct and unusual (Alserhan, 2010; Havinga, 2010). One of the most common misconceptions arising from Islamophobia is that consuming halal products means promoting Islam and exclusively helping Muslims’ economies (Rios et al., 2014). Misunderstandings based on incorrect beliefs, insufficient awareness, misinformation, or confusion can lead to boycott behavior. Boycotts, particularly religion-based boycotts, can be long-lasting and destructive, specifically in the marketing sector, because they usually involve deeply held personal views (Abosag and Farah, 2014; Muhamad et al., 2019). Throughout various non-Muslim nations, the anti-halal agitation developed from misunderstandings concerning Islam or Islamophobia, leading to a boycott of halal products (Hussein, 2015). Social media has become the primary means of communication for many Indians searching for new knowledge. As a result, anything, especially anti-halal propaganda, spreads quickly. Negative knowledge or sensationalized topics are promptly conveyed and spread without reservation. This may have a negative impact on consumer attitudes about halal items. In India, the boycotting of halal products is at an all-time high, resulting in a great deal of hostility and a lack of buying intentions toward halal products. We propose the following hypothesis based on the above reasoning:

*H6a*: Consumer animosity mediates the relationship between boycott attitude and unwillingness to buy halal products.

If individuals have animosity against a specific religion, ethnocentric consumers usually avoid buying things from that faith. Because of their hostility against particular faith, ethnocentric individuals will demonstrate negative behavior toward the products. Ethnocentric individuals believe that their religious beliefs are of superior value and that goods produced by people of other faiths are of lower value. According to the previous research, ethnocentrism has a detrimental impact on consumer purchasing intentions (Abosag and Farah, 2014). Consumers with an ethnocentric viewpoint will choose to purchase local goods from their particular nation (hereunder non-halal products) due to a sense of patriotism to their particular country, religious affiliation, or a feeling of betrayal if they purchase halal products (Sharma, 2015). We suggest the following hypothesis based on the preceding arguments:

*H6b*: Consumer animosity mediates the relationship between consumer ethnocentrism and unwillingness to buy halal products.

Klein et al. (1998, p. 90), emphasizing buyers, describe consumer animosity as “remnants of aversion associated with prior or ongoing military, political, or economic events.” Consumer prejudice is strongly tied to consumer animosity. According to Ouellet (2007, p. 115), consumer prejudice is “the aversion against a specific ethnic minority’s products or services as a representation of discrimination against that group.” Klein et al. (1998) show that animosity is linked to a lower desire to buy goods from the central ethnic group (hereunder halal vendors). Based on the above observations and concepts, it seems that animosity, instead of consumer prejudice, is the initial step. A person who has a strong affiliation with a country does not have to be unfriendly to other cultures; however, some people who strongly identify with a country possess resentment toward other cultures (Rawwas et al., 1996). Such people may see the arrival of new goods from a different culture as an invasion of a minority culture that threatens to damage the domestic culture. Individuals with a significant sense of patriotism regard their religious faith as superior to other religions. We suggest the following hypothesis based on the previous concepts and the long, tumultuous history of disputes between Indian Muslims and Hindus.

*H6c*: Consumer animosity mediates the relationship between patriotism and unwillingness to buy halal products.

**Figure 1:**
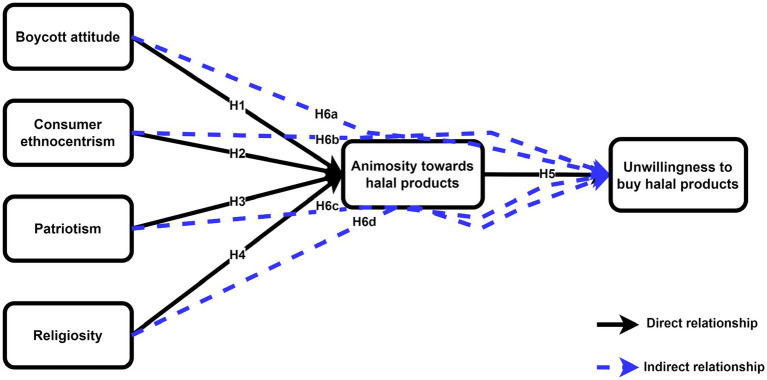
Conceptual framework of the study.

The term “religiosity” refers to a person’s individual belief in God mixed with a determination to adhere to specific God-given principles (Mortimer et al., 2020). Religion has a significant impact on people’s ideas and values, as well as on how they evaluate marketing and goods, and therefore, religion influences their intake and purchasing intentions (Fam et al., 2004; Farah and El Samad, 2014). As a result, very religious people are inclined to think about the world around them through their religious beliefs. This means that they act differently than people who are less religious (Worthington et al., 2003). Béji-Bécheur et al. (2012) find that everyone has a variety of identities, including familial, religious, local, and national identities. Religious identification is one of the most significant characteristics because, in contrast to spiritual demands, faith and religious bodies provide societal, economical, and physiological advantages (Peek, 2005). Religion is a significant meaning-making system that aids people in managing existential anxiety and making sense of their lives (Greenberg et al., 1997). Based on this, we feel that the religious attitude against halal items in the Indian Hindu setting is at an all-time high, resulting in hatred and a lack of purchasing intention (Figure 1). We suggest the following hypothesis based on the preceding arguments:

*H6d*: Consumer animosity mediates the relationship between religiosity and unwillingness to buy halal products.

## Methodology

This study focuses on India Hindu consumers and analyzes the questionnaire responses of 512 Indian research participants who regularly purchase fast-moving consumer goods. The study follows Brus and De Gruijter (2003) recommendation of non-probability criterion-based convenience sampling because it allows us to explore respondents’ experiences with the main phenomenon. We create a questionnaire by editing it in English, then translating it into Hindi using the back-translation procedure with a preliminary trial of 15 participants. The questionnaire is then modified according to feedback. The questionnaire is uploaded to Microsoft Forms,^1^ an online platform that offers survey structure, circulation, management, and scrutiny facilities. The data used for analysis were collected from January 2021 to April 2021. Before beginning the survey, we present our participants with an information sheet about the study that guarantees their anonymity and explains the current study goals.

### Sample Profile

Sixty-two percentage of the participants are male, 32% female, and the remaining 6% prefer not to say. All the participants are of Indian nationality, with 79% residing in India, and 21% living in another country. Six percentage of the participants are below the age of 20, 49% are aged 21–30, 16% are aged 31–40, 19% are 41–50 years old, and only 10% are over 50 years old. Totally participants are employed or studying in India at the time of the study (though, as mentioned above, some have official residence elsewhere). Sixty-nine percentage of the total participants are employed, with the remaining 31% being unemployed. Fifty-three percentage hold undergraduate qualifications, 35% hold postgraduate qualifications, and the remaining 12% hold a doctorate.

### Construct Measurement

The variables are assessed employing five-point Likert scores ranging from “strongly disagree” (scored as one) to “strongly agree” (scored as five). All the research items are adapted from the available literature and revised to the framework of this research. The sources of the constructs are as follows: boycott attitude (Klein, 2002), religiosity (Ahmed et al., 2013), patriotism (Marinkovic, 2017), consumer ethnocentrism (Klein, 2002; modified from Shimp and Sharma, 1987), animosity toward halal products (Ayyub, 2015), and unwillingness to buy halal products (Rose et al., 2009). Each of the variable items has been modified to fit the needs of this research.

### Analyses

We perform the appropriate data filtering operations before initiating any analysis to verify data entry accuracy and evaluate the normalcy of continuous parameters. Missing values are filled with the mean of the subscale’s items. Secondly, the data are analyzed for outliers, which are described as deviations from the sample mean of more exceed 3.5 SDs for every construct (Hair et al., 2013). Third, we run the conceptual framework with partial least squares (PLS) evaluation using SmartPLS (v.3.2.7) application (Ringle et al., 2015). Fourth, we conduct a consistent PLS bootstrapping procedure with replacement using 5,000 subsamples to calculate the parameter estimates’ statistical significance. We also conduct a PLS Multi-Group Analysis (PLS-MGA) to test whether the model shows a significant difference between male and female group-specific parameter estimates (Sarstedt et al., 2011), and find no significant difference.

### Measurement Model

#### Common Method Bias

Common method bias (CMB) might exist because the current study predictor variables are represented by a similar responding method (Podsakoff et al., 2003). Many researchers (Schwarz et al., 2017; Rodríguez-Ardura and Meseguer-Artola, 2020; Kock et al., 2021) propose various precautions to regulate CMB, such as participant anonymity, trying to avoid ambiguous research questions, and offering detailed guidance in surveys to reduce bias and glitch. In contrast to these measures, we use a modern approach and evaluate CMB by analyzing collinear constructs and associated items (Rodríguez-Ardura and Meseguer-Artola, 2020). According to our findings, the CMB is not a problem while evaluating the structural model because the VIF values are below 5 (Table 1). The inter-construct correlation, as proposed, is another way to test the CMB (Bagozzi et al., 1991). In this analysis, the inter-construct correlation should be below 0.90; Table 2 shows the statistics of inter-construct correlation, where all the numbers are below 0.90. As a result, CMB is not a problem when it comes to evaluating the structural model.

**Table 1:** Factor loadings (FL), Cronbach alpha (*α*), composite reliability (CR), Dijkstra–Henseler’s rho (rho A), average variance extract (AVE), and variance inflation factor (VIF).

Constructs with items	FL	*α*	rho A	CR	AVE	VIF
**Boycott attitude**		**0.889**	**0.890**	**0.931**	**0.818**	
By boycotting halal products, I can change the halal products business in India.	0.890					2.372
Everyone should take part in boycotting halal products in India.	0.925					3.027
My friends/family should support me to boycott halal products in India.	0.898					2.565
**Consumer ethnocentrism**		**0.918**	**0.926**	**0.949**	**0.862**	
It is not right to purchase halal products because it puts Muslims out of jobs.	0.854					2.110
A real Hindu Indian should always buy non-halal products.	0.971					3.170
We should always purchase non-halal products instead of letting Muslim businessmen get rich off us.	0.956					4.120
**Patriotism**		**0.874**	**0.878**	**0.923**	**0.799**	
As an Indian Hindu, I am proud to be a citizen of India.	0.889					2.571
As an Indian Hindu, I am emotionally attached to India.	0.892					2.224
As an Indian Hindu, I am proud to live in India.	0.894					2.342
As a Hindu, I am proud to see the national flag wavering.^*^						
**Religiosity**		**0.842**	**0.847**	**0.894**	**0.679**	
I go to the temple (place of worship) regularly.	0.758					1.731
Spiritual values are more important than material things.	0.804					1.956
If Muslims respect the Hindu religion, this would be a better religion.	0.824					2.276
I consider myself to be very religious.	0.846					2.358
**Animosity toward halal products**		**0.867**	**0.867**	**0.898**	**0.657**	
I dislike halal vendors.^*^						
I have negative view about halal products.	0.661					1.399
Halal vendors want to gain economic power over non-halal businesses.	0.740					2.910
I feel angry toward Muslims for the terrorist acts they have committed.	0.775					4.233
I felt fear when I saw the Arabic halal stamp on products.	0.765					3.860
Muslim businesses cannot be trusted.	0.728					2.280
Muslim business people are taking advantage of the rest of us.	0.781					2.675
Muslims do business unfairly with other non-Muslims.	0.769					2.433
**Unwillingness to buy**		**0.859**	**0.862**	**0.899**	**0.640**	
I would feel guilty if I bought halal products.	0.790					2.157
I would never buy halal products.	0.787					2.152
Whenever possible, I avoid buying halal products.	0.819					2.090
I do not like the idea of owning halal products.	0.784					1.842
If two products were equal in quality, but one was halal, and one was not, I would pay 10 percent more for the non-halal product.	0.819					2.080

* Items dropped due to factor loading less than 0.6. The bold values are the square root of AVE.

**Table 2:** Discriminant validity and model fit.

C#	1	2	3	4	5	6	SRMR	NFI	GOF
**Fornell and Larker method**									
1	**0.904**								
2	0.358	**0.928**							
3	0.637	0.619	**0.747**						
4	0.302	0.403	0.429	**0.894**					
5	0.508	0.654	0.713	0.463	**0.824**				
6	0.499	0.574	0.757	0.572	0.659	**0.800**			
**HTMT method**									
1	**0.000**								
2	0.394	**0.000**							
3	0.731	0.685	**0.000**						
4	0.341	0.447	0.487	**0.000**					
5	0.585	0.748	0.826	0.534	**0.000**				
6	0.570	0.643	0.671	0.656	0.771	**0.000**			
Model fit							0.093	0.724	0.807

1 = boycott attitude, 2 = consumer ethnocentrism, 3 = animosity toward halal products, 4 = patriotism, 5 = religiosity, 6 = unwillingness to buy. The bold values are the square root of AVE.

#### Internal Consistency

There are two key indicators of internal consistency: Cronbach’s alpha and the overall reliability of the overall survey process. Hair et al. (2016) determined that an alpha value between 0.60 and 0.70 is appropriate. Cronbach’s alpha ranges between 0.842 and 0.918, as indicated in Table 3. Composite reliability, or construct reliability (CR), refers to the internal consistency of components that have been altered to fit particular constructions (Henseler et al., 2009). The accepted standard value for CR is 0.60 at the very least (Fornell and Larcker, 1981a). Table 1 demonstrates that the measuring scale’s internal consistency ranges between 0.894 and 0.949.

**Table 3:** Coefficient of determination (*R*^2^), effect size (*f*^2^), and predictive analytics (*Q*^2^).

Construct	Animosity toward halal products	Unwillingness to buy
*R* ^2^	0.647	0.573
*Q* ^2^	0.319	0.335
*f* ^2^		
Boycott attitude	0.266	
Consumer ethnocentrism	0.092	
Patriotism	0.009	
Religiosity	0.156	
Animosity toward halal products		1.344
Unwillingness to buy		

#### Construct Validity

Content validity, convergent validity, and discriminant validity are measured by factor loading (Fornell and Larcker, 1981b; Hair et al., 1998; Chin et al., 2003). Discriminant validity is demonstrated when the average variance extract (AVE) square root is larger than the inert-item associations. With the Fornell and Larcker and HTMT approach, this research meets Fornell and Larcker (1981a) criteria for proving discriminant validity (see Table 2).

### Structural Model

#### Model Fit

The standardized root means residual (SRMR) value (Table 2) of this study is 0.093, which, being less than 0.1, represents a good model fit (Hu et al., 1998; Henseler et al., 2014). According to the definition, the normed fit index (NFI) equals one less than the proposed model’s Chi-square value divided by the Chi-square values. As a result, the NFI’s values range from 0 to 1. The greater the fit, the nearer the NFI is to 1. NFI values greater than 0.9 generally indicate a good match (Lohmöller, 1990). The proposed model’s NFI result is 0.724, which is a respectable number (Table 2). The existing research additionally calculates the goodness-of-fit (GOF) value, as recommended by Tenenhaus et al. (2005), to assess and evaluate the efficiency of the proposed research. The following formula can be used to determine the GOF:


$$GOF=\sqrt{\bar{AVE}\times {\bar{R}}^{2}}$$

The GOF is proven based on the computed value of 0.807 (Table 2). It is increasingly significant and above the predefined threshold value of 0.36 (Wetzels et al., 2009).

#### Coefficient of Determination (*R*^2^), Effect Size (*f*^2^), Predictive Analytics (*Q*^2^), and Importance-Performance Map Analysis

As a general rule, the value of *R*^2^ for endogenous construct denotes 0.75, 0.50, and 0.25 as substantial, moderate, and weak, respectively (Henseler et al., 2016; Sarstedt et al., 2017). In this research, the value of *R*^2^ for consumer animosity is 0.647 and the unwillingness to buy is 0.573. Rather than relying just on *R*^2^, researchers suggest Stone–Geisser (*Q*^2^) as a more effective indicator of the model’s analytical usefulness (Sarstedt et al., 2017). The blindfolding technique determines *Q*^2^, representing how accurately the path model accurately anticipates the actual observable values. Accordingly, small, medium, and high predictive importance is represented by the *Q*^2^ values of 0.02, 0.15, and 0.35 (Sarstedt et al., 2017). Our model’s *Q*^2^ for customer animosity is 0.319, and its value for reluctance to purchase is 0.335, indicating that it is highly predictive. In addition to *R*^2^ and *Q*^2^, the researchers also calculate the effect size (*f*^2^). The *f*^2^ statistic is designed to assess the impact of the latent variable on the endogenous components. The *f*^2^ values may be classified as small, medium, or big, with 0.02, 0.15, and 0.35 being the best (Cohen, 1988). The *f*^2^ values are all excellent, as per our results reported in Tables 3, 4.

**Table 4:** Importance-performance map analysis.

Construct	Total effects	Performances
Boycott attitude	0.269	75.195
Consumer ethnocentrism	0.181	80.737
Patriotism	0.181	83.862
Religiosity	0.262	80.850
Animosity toward halal products	0.757	79.724

**Figure 2:**
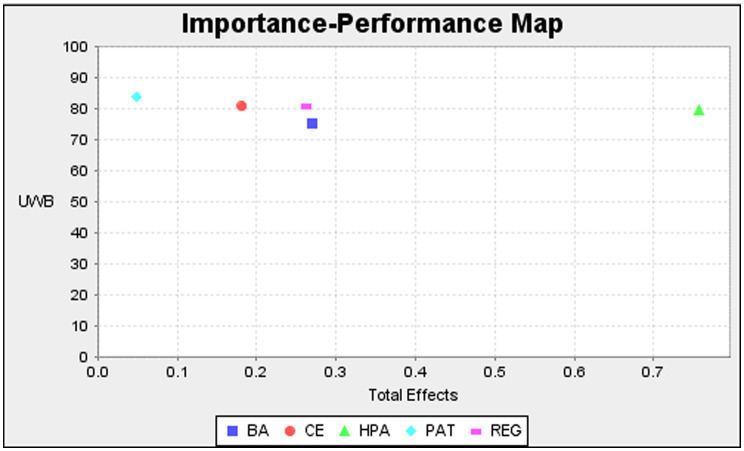
Importance-performance map analysis (IPMA).

The basic purpose of importance-performance map analysis (IPMA) is to determine which determinants possess high performance but the low significance and vice versa (Sarstedt et al., 2017). IPMA values indicate that boycott attitude, consumer ethnocentrism, patriotism, religiosity, and consumer animosity have importance values of 0.269, 0.181, 0.049, 0.262, and 0.757, and performance values of 75.195, 80.737, 83.862, 80.850, and 79.724, correspondingly. If we equivalenced the values, we can see that all the independent variables for unwillingness to buy halal products by Indian consumers ranged between 75 and 85, thereby differing slightly. However, patriotism contributed more than other constructs in strengthening the unwillingness to buy, followed by religiosity (Figure 2).

#### Hypothesis Testing

The path coefficients (*β*) and significance (*p*) values are calculated in the structural model. We may use this approach to examine whether hypotheses are supported or not supported based on their results. On the basis of Table 5 findings, the positive effect of boycott attitude on consumer animosity (*β* = 0.356, *p* < 0.001), the positive effect of consumer ethnocentrism on consumer animosity (*β* = 0.240, *p* < 0.001), the positive effect of patriotism on consumer animosity (*β* = 0.065, *p* > 0.050), the positive effect of religiosity on consumer animosity (*β* = 0.346, *p* > 0.05), and the positive effect of consumer animosity on the unwillingness to buy halal products (*β* = 0.757, *p* < 0.001) support hypotheses H1, H2, H4, and H5, while H3 is rejected.

**Table 5:** Path coefficient results.

Hypotheses	Relationship	Path coefficients	T statistics	*p*	Decision
**Direct effects of constructs**					
H1	Boycott attitude → animosity toward halal products	0.356	7.891	0.000	Supported
H2	Consumer ethnocentrism → animosity toward halal products	0.240	4.434	0.000	Supported
H3	Patriotism → animosity toward halal products	0.065	1.427	0.154	Not supported
H4	Religiosity → animosity toward halal products	0.346	5.489	0.000	Supported
H5	Animosity toward halal Products → unwillingness to buy	0.757	17.860	0.000	Supported
**Indirect effects of animosity toward halal products (HPA)**					
H6a	Boycott attitude → HPA → unwillingness to buy	0.269	7.141	0.000	Supported
H6b	Consumer ethnocentrism → HPA → unwillingness to buy	0.181	4.283	0.000	Supported
H6c	Patriotism → HPA → unwillingness to buy	0.049	1.414	0.157	Not supported
H6d	Religiosity → HPA → unwillingness to buy	0.262	5.202	0.000	Supported

**Figure 3:**
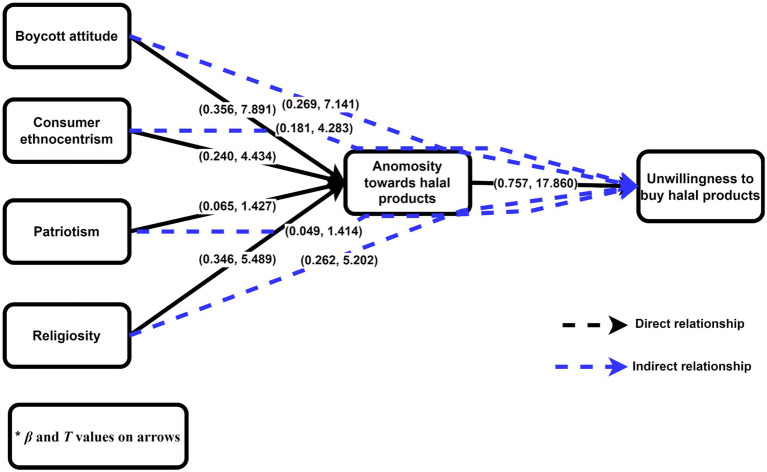
Path coefficient results of the study.

As Zhao et al. (2010) suggested, partial mediation exists if the direct effect and mediation effect are positive. Based on this recommendation, we can say partial mediation exists. In addition, if the direct effect is insignificant and the mediation effect is significant, then full mediation exists. When the direct effect is significant and the mediation effect is insignificant, no mediation exists. We find that boycott attitude has a direct positive effect on the unwillingness to buy halal products (*β* = 0.142, *p* < 0.01), and consumer animosity mediates the positive relationship between boycott attitude and the unwillingness to buy halal products (*β* = 0.269, *p* < 0.001), supporting the hypothesis H6a. We find complementary partial mediation exists. Consumer ethnocentrism has an insignificant effect on the unwillingness to buy halal products (*β* = 0.025, *p* > 0.05), while consumer animosity mediates the positive relationship between consumer ethnocentrism and the unwillingness to buy halal products (*β* = 0.181, *p* < 0.001), supporting the hypothesis H6b. We find full mediation exists. Patriotism has a direct positive effect on the unwillingness to buy halal products (*β* = 0.092, *p* < 0.001), and consumer animosity mediates the insignificant relationship between patriotism and unwillingness to buy halal products (*β* = 0.049, *p* > 0.050), rejecting the hypothesis H6c. We find no mediation exists. Religiosity has an insignificant effect on the unwillingness to buy halal products (*β* = 0.032, *p* > 0.05), while consumer animosity mediates the positive relationship between religiosity and unwillingness to buy halal products (*β* = 0.262, *p* < 0.001), supporting the hypothesis H6d. We find full mediation exists (Figure 3).

## Discussion

This research is designed to provide a more comprehensive understanding of the role of consumer animosity in mediating the relationship between boycott attitudes and religiosity, patriotism, and consumer ethnocentrism, respectively. Further, this study aims to elucidate the relationship between boycott attitudes mediated by consumer animosity and consumers’ unwillingness to buy halal products. The framing of the current research contributes significantly to animosity literature. This study represents an effort to step away from previous findings on consumer animosity and its effect on buying behavior. Several researchers on buying behavior (Kumar and Devi, 2018; Prabha, 2018) attempt to understand the relationship between consumer animosity toward halal products and the unwillingness to buy halal products, and various proposed animosity frameworks have empirically proven that significantly positive animosity factors exist. In the context of India, our results validate the current market animosity. This study’s uniqueness sheds light on the context of religiosity, patriotism, and consumer ethnocentrism, which leads to boycott attitudes against halal products and can explain the reluctance of consumers to buy such products.

To the best of our knowledge, consumer animosity has not been previously investigated as a performative factor that can ruin a person’s feelings toward products. This study also contributes to the existing research on the COO effect by illustrating consumer animosity’s influence regarding halal products. This study aims to fill a knowledge gap by demonstrating how animosity toward halal products plays a crucial role between different religions. Grounding the role of four constructs (boycott attitudes, religiosity, patriotism, and consumer ethnocentrism) in the Indian context, this research has expanded our understanding of consumer animosity. Indian consumers with high levels of patriotism and religiosity tend to feel more animosity toward halal products. Our results indicate that consumer animosity influences the reluctance to purchase halal products. Moreover, history should not overlook ordinary people. A religious dispute between Indian Hindus and Muslims and the Indian political leaders and influencers’ boycott initiative shows that this animosity toward halal products or Muslims persists at the interpersonal, political, and societal levels. For instance, under Prime Minister Narendra Modi’s leadership, Indian political leaders have publicly announced that India should be considered “*Ram Rajya*” or “*Hindustan*,” impacting Indian (Hindus) consumers’ unwillingness to buy halal goods.

In short, these findings extend past research within each social group on market ethnocentrism and animosity. The study reflects on a continuous and relatively high animosity framework and directly explores and identifies acceptance throughout population groups. In the intensity of animosity toward halal products or Muslims, significant differences can exist between social groups. Our exploratory study shows that customers can create an extremist level of animosity against halal products for various reasons. Our research therefore highlights two main issues. First, contemporary events (for example, the ongoing religious conflict and international terrorism) are more present in the minds of consumers than historical events. Therefore, it is justifiable that market animosity influences customers’ purchasing behavior (Klein, 2002). Customers’ negative feelings can detrimentally affect their willingness to purchase goods associated with the “enemy” nation, thereby contributing to animosity. Second, whereas current ongoing problems play an important role as drivers of animosity, many additional constructs, such as religiosity, substantially affect individuals’ animosity toward halal products.

The findings of the current study further indicate that there is a positive link between ethnocentrism and animosity. More nationalistic and conservative Indian customers usually show high levels of ethnocentrism, which contributes to a positive effect on hostility toward Muslims. This finding is in line with those of Nijssen and Douglas (2004). However, the present research also shows that ethnocentrism increases halal product animosity, and it significantly affects the reluctance to buy halal products. Among Indian consumers, nationalist emotions constrain them from purchasing halal products. Moreover, the feeling of animosity resulting from persistent religious conflicts, political strife, and economic woes has increased the reluctance to buy halal products (Narang, 2016; Shoham and Gavish, 2016).

### Managerial Implications

These results have consequences for policymakers seeking to resolve the preconception of consumer animosity toward halal products. Let us suppose that business leaders in halal firms wish to reduce animosity levels. Their first step should be to recognize and respect individual citizens’ emotions according to the country’s religion and maintain a professional business image with the nation at the federal level. Policymakers must also consider the impact of patriotism and religiosity among the nation’s citizens. By demonstrating that the effects of animosity toward halal products are much more significant than other variables, our study offers reasonable solutions to enhance economic and societal cooperation between majority and minority citizens. Although it is often accepted that friendly relations in society will promote economic cooperation (Makino and Tsang, 2011; Li et al., 2019), our findings show that it is far more necessary to decrease animosity among ordinary people than to endorse marketing campaigns in the nation.

Occasional economic rivalry is to be expected as one of the consequences of globalization. For any country, nationalism becomes one of the most significant factors in promoting its products to its citizens. For example, Patanjali is a famous brand for Indian consumers, especially Hindus. Even political party leaders in India have recently criticized halal products and urged Indian people to boycott halal products and services. Thus, halal marketers should be aware of such consequences when entering or working in the Indian market. When animosity is mainly characterized by anger and hatred, as in the case of the Hindus and Muslims who clash in India, the appropriate approach would be to set as much distance as possible between a brand and its COO. Companies can also select joint ventures and market their products with regional stakeholders to advertise their goods (Fong et al., 2014). If positive, this approach will reduce the amount of retaliation and raise consumers’ stakes in the organization’s business. As consumer ethnocentrism positively affects consumers’ unwillingness to buy, marketing professionals should highlight the importance and benefits of their products by, for example, establishing partnerships for joint product production within the marketing economy.

Overall, while emphasizing the role of religious conflict in India and Hindu elites’ calls to boycott, we recognize that the policymaker of the halal products industry should manage and resolve their local campaigns to save their reputation. Moreover, through this research, halal food marketers can gain some knowledge to maximize their profits and develop India as a hub of the halal industry. These findings can facilitate an understanding of the purchase intentions of non-Muslims toward halal products for both academics and the food industry.

### Limitations and Future Research Directions

The most significant limitation of the study is that generally speaking, our analysis is exploratory. Our research supports the notion that consumer animosity plays a vital role in the nationwide unwillingness to buy halal products. Under the consideration of four variables (boycott attitude, patriotism, religiosity, and ethnocentrism), our analysis demonstrates that, perhaps unsurprisingly, animosity tends to be an ever-present nationwide reality. Our findings reveal a direct connection between consumer animosity and the unwillingness to buy halal products. Using our results, we can collect more regional data and determine the full impact of consumer animosity. Although our analysis examines how the existence of animosity at the national level can be described, more intricate methods to explore this animosity should be pursued in future studies. For example, our study uses sample data from North India (Delhi and NCR region), and future research could expand upon this region (Rizkitysha and Hananto, 2020).

Furthermore, India has a highly diverse culture, which is one of the most challenging obstacles to representing national animosity. There are substantial variations in national animosity in India, with different degrees of tension specific to particular regions (Gao et al., 2017). A uniform program may be productive to the degree that the products themselves come from a nation where animosity is minimal or regular throughout short- or mid-term periods. However, levels of animosity may vary by population, as is the case in this study. High levels of animosity often influence consumer decisions. Marketers, therefore, need to understand, monitor, and discuss particular attitudes across social groups. In addition, counterculture and social identity can be more significant factors in animosity than national identification. The importance of counterculture in the evaluation of halal products has been alluded to in this research. However, further research is needed to investigate the reasons, sources, and repercussions of animosity throughout demographic variables and cultural groups.

Furthermore, a high standard of work should be implemented based on customers’ explanations rather than following the Klein et al. (1998) scale as one that is universally implemented. Finally, further support should be given to a study of context-specific animosity. With this intention, observational consumer surveys could disclose the deeper causes of animosity expressions to ensure the motivations’ completeness and consistency, thus appealing to various internal reliability stages.

## Data Availability Statement

The raw data supporting the conclusions of this article will be made available by the authors, without undue reservation.

## Author Contributions

FA: study conception and design. MF: data collection. FA, MT, and PZ: analysis and interpretation of results. MT, EL-Q, and JA: draft manuscript reviewing, editing, and supervision. All authors reviewed the results and approved the final version of the manuscript.

## Funding

The authors thank the Spanish Ministry of Science and Innovation (PID2020-113469GB-I00), the Junta de Castilla y León and the European Regional Development Fund (grant CLU-2019-03) for the financial support to the Research Unit of Excellence “Economic Management for Sustainability” (GECOS) and the National Natural Science Foundation of China (中国国家自然科学基金项目; grant no. 项目批准号: 72072026).

## Conflict of Interest

The authors declare that the research was conducted in the absence of any commercial or financial relationships that could be construed as a potential conflict of interest.

## Publisher’s Note

All claims expressed in this article are solely those of the authors and do not necessarily represent those of their affiliated organizations, or those of the publisher, the editors and the reviewers. Any product that may be evaluated in this article, or claim that may be made by its manufacturer, is not guaranteed or endorsed by the publisher.

## Footnotes

^1^ https://forms.office.com/
